# Geographical dataset of firearms manufacturing in the United States: 2000–2020

**DOI:** 10.1016/j.dib.2022.108626

**Published:** 2022-09-21

**Authors:** Lisa Jordan, Marwa Elessawy, Graham Munro-Ludders

**Affiliations:** Drew University, 36 Madison Ave., Madison NJ 07940, USA

**Keywords:** Firearms, Production, Guns, Locations

## Abstract

Annual Firearms Manufacturing and Export Reports (AFMER) published annually by the United States Bureau of Alcohol, Tobacco, Firearms and Explosives (ATF) were digitized, cleaned, merged, aggregated, and georeferenced to produce a geographical time-series dataset. By preserving unique identifiers and locational information, the geographical dataset of firearms manufacturing enables quantitative research across public health data records, including compliance reporting by U.S. Occupational Safety and Health Association, Environmental Protection Agency, and Department of Commerce.

The dataset includes records for 8,707 unique manufacturing licensees, with a total of 133,952,899 firearms released into commerce between 2000 and 2020. Records include the latitude and longitude attributes for manufacturing locations that produced more than one percent of firearms released into commerce during the study timeframe (n=100). To facilitate research, the dataset was stored in multiple formats, including table formats, such as comma-separate values and excel format, as well as GIS shapefile format.

The data may be explored in multiple dimensions: by time, by location, and by type of firearm released into commerce. Types of firearms include: miscellaneous firearms, pistols (by caliber: 22, 25, 32, 38, 50, 9mm), rifles, revolvers (by caliber: 22, 32, 35, 38, 44, 50), and shotguns. Total firearms manufactured by year and in aggregate and total exports, by firearm type, and by year were calculated. Percentage of total firearms manufactured for each location was also determined. The dataset does not include used firearms or firearms imported to the United States.

Several software packages enabled the production of this dataset. Adobe Acrobat software was used to convert archival .PDF files into comma-separated value tables. The authors systematically verified and validated the conversion process. Annual tables were merged by the unique identifier, RDS Key, across time periods, using Stata statistical software. Aggregation took place in Stata and Excel software, and percent of total firearms manufactured was calculated for each location. Aggregate tables identifying locations in descending order for total reported firearms released into commerce (2000-2020) were generated in Excel.

ArcGIS Online software was used to geocode manufacturing locations where more than one percent of total study firearms were produced. ArcGIS Pro software was used to identify centroid, or latitude and longitude locations for the facilities. ArcGIS Pro software was also used to create a summary figure, and GIS shapefile feature data.

**Specifications Table**

**Table:** 

Subject	Public Health and Health Policy.
Specific subject area	Firearms
Type of data	Table Figure GIS vector feature data
How the data were acquired	The U.S. Bureau of Alcohol, Tobacco, Firearms and Explosives (ATF), a branch of the U.S. Department of Justice, reports annually on the number of firearms sold by manufacturers by firearm type (pistol, rifle, shotgun, revolver, or miscellaneous). Included in each report are total numbers of firearms by type sold into commerce by federal firearms licensed (FFL) manufacturers, including the license name and street address. Specifically, each record in the Annual Firearms Manufacturing and Export Report table represents a uniquely licensed manufacturing location. Data for 2007-2020 were available from the ATF website from the *Annual Firearms Manufacturing and Export Report* (AFMER). From ATF-hosted copies, as well as previously published reports archived by the Homeland Security Digital Library and GitHub (2000-2006), the collected PDF documents were converted into spreadsheets using Adobe Acrobat software. The individual spreadsheets were then merged across different firearm types using the RDS (region-district-sequence) key, a unique identifier for each gun manufacturing location, for each year, using Stata statistical software. Multiple years were appended by RDS keys to create a time series panel data for 2000-2020. The wide form of the panel data set records 8,707 federal firearms license manufacturing locations.
Data format	Raw Analyzed Filtered
Description of data collection	Once the raw data were aggregated across the study time period, 2000-2020, the data were sorted by total firearms manufactured. The manufacturing locations that produced more than one percent of total manufactured firearms were selected (n=100). These locations were georeferenced using ArcGIS Online geocoding services, and imported into ArcGIS Pro software. Centroid coordinates were appended to a vector database, and exported in GIS shapefile format.
Data source location	[1] U.S. Bureau of Alcohol, Tobacco, Firearms and Explosives. Data & Statistics [Internet]. Data & Statistics | Bureau of Alcohol, Tobacco, Firearms and Explosives, Annual Firearms Manufacturers and Export Report (2007-2020). 2022 [cited 2021 Jun 03]. Available from: https://www.atf.gov/resource-center/data-statistics [2] U.S. Bureau of Alcohol, Tobacco, Firearms and Explosives. Annual Firearms Manufacturers and Export Report (2000, 2002, 2003) U.S. Center for Homeland Defense and Security. Homeland Security Digital Library. Homeland Security Digital Library. 2021 [accessed 2021 Jun 11]. https://www.hsdl.org/?search=&searchfield=&all=annual+firearms+and+export+report&collection=public&submitted=Search [3] U.S. Bureau of Alcohol, Tobacco, Firearms and Explosives. Annual Firearms Manufacturers and Export Report (2001, 2004-2006), Distributed by Jones R. ryjones/AFMER. 2020. https://github.com/ryjones/AFMER/tree/default/SOURCE/NU
Data accessibility	Repository name: Mendeley Data [4] Data identification number: DOI: 10.17632/gmz374n75n.2 https://doi.org/10.17632/gmz374n75n.2 Direct URL to data: https://data.mendeley.com/datasets/gmz374n75n/2

## Value of the Data

• This dataset provides information on the major sources for the supply and the quantity of firearms in the U.S., by firearm type, annually, from 2000-2020.
• Public health research involving gun safety and gun violence studies can benefit from an improved understanding of the quantity and locations of firearms produced in the United States.
• International studies and human rights research concerned with firearms exports from the U.S. benefits from access to aggregate information, by time and place, on licensed manufacturers that produce and distribute firearms for export.
• Changes in type, quantities, and locations of firearms produced over time can be explored with this dataset. Also, industry concentration in firearm production can be studied.
• Georeferenced datasets with unique identifiers are suitable for joining and merging with other databases. For example, time series AFMER records can be joined with EPA compliance databases and OSHA inspection databases to study patterns in inspections and compliance over geographic regions. Both of which can indicate changes in compliance over time.
• Georeferenced time-series data sets enable research on social and demographic characteristics of communities where manufacturing takes place.

## 1 Data Description

**Fig. 1:**
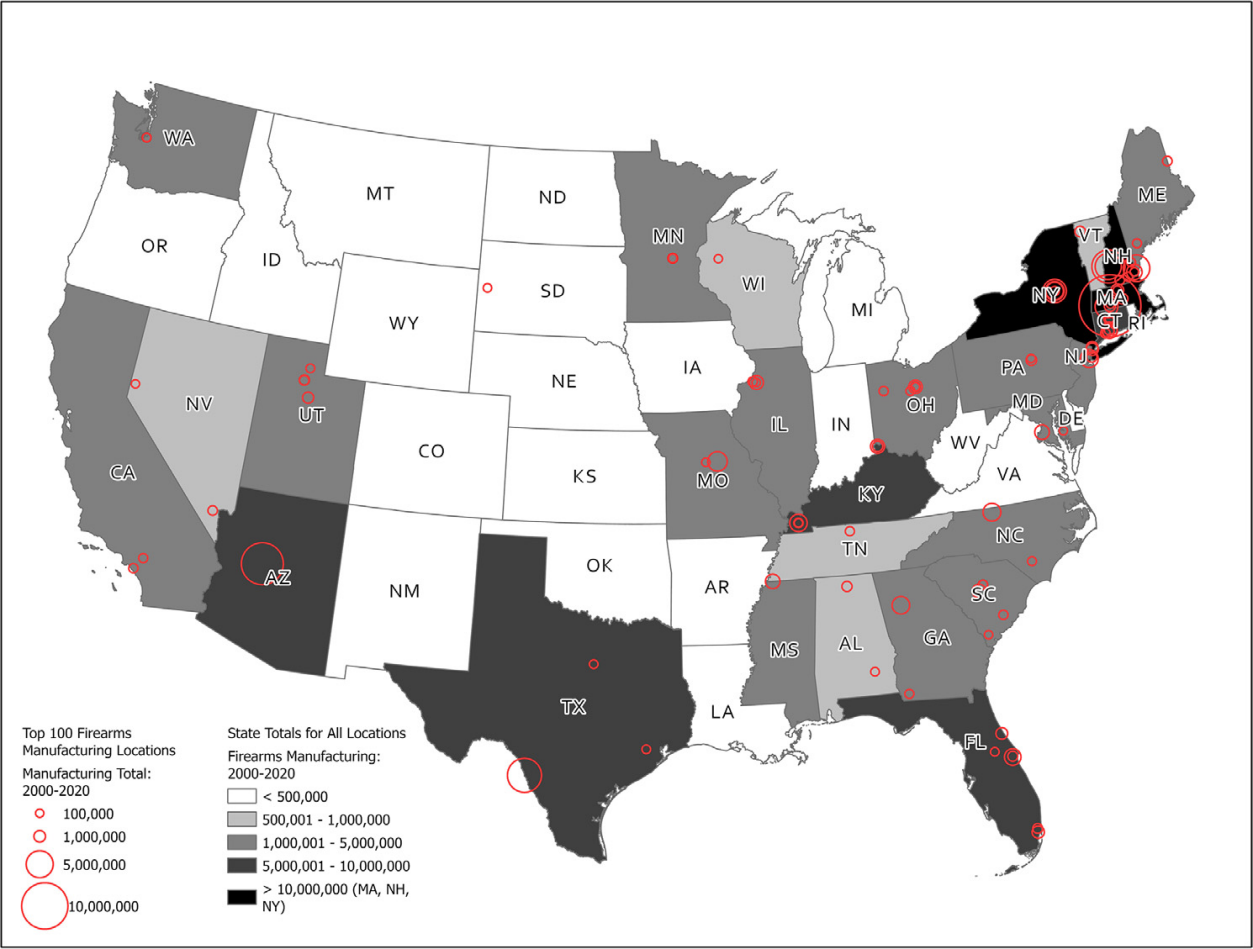
Top Firearms Manufacturing Locations, and Total State Production, 2000–2020.

The dataset contains three, downloadable files: AFMER Time Series 2000-2020 – Wide.xlsx (Excel file), AFMER Time Series 2000-2020.csv (comma-separate values), and AFMER_2000_2020.zip (zipped GIS Shapefile). The AFMER Time Series 2000-2020 – Wide.xlsx contains five sheets. The first sheet contains a comprehensive list of unique RDS Keys, names of federal firearms licensees (FFL), locations for the licensed manufacturers, the types of firearms manufactured, or exported, by year, as well as aggregation variables for manufacturing and exports from 2000-2020. The second sheet lists the U.S. states and the total firearms manufactured in each state from 2000-2020. The third sheet lists a selection of the first sheet, displaying firearms manufacturing locations that contributed more than one percent to total firearms manufactured from 2000-2020. There were 100 records with manufacturing locations that contributed more than one percent to total firearms manufactured. The fourth sheet is a table of the top firearms manufacturing locations that produced, in total, 75 percent of all firearms manufactured, from 2000-2020. The fifth sheet lists the top firearms manufacturers, aggregating their production across multiple locations. This manufacturing list accounts for 80 percent of firearms manufactured from 2000-2020. The last sheet, also displayed in Fig. 1, includes a .GIF figure illustrating the locations listed in the second sheet (‘State Totals’) and third sheet (‘AFMER Time Series – Top 100’).

The .CSV file includes the first sheet in AFMER Time Series 2000-2020.xlsx. The AFMER_2000_2020.zip includes a vector, GIS Shapefile. This file can be displayed using GIS software to show the locations of manufacturers included in the ‘AFMER Time Series – Top 100’ list. Also included in the Shapefile are the X and Y coordinates for the manufacturing locations, as georeferenced with ArcGIS Online.

## 2 Experimental Design, Materials and Methods

First, contemporary and archival records of the Annual Firearms Manufacturing and Export Report were obtained. From 2000-2019, these data were released in .PDF format. Individual .PDF files were converted to spreadsheet format using Adobe Acrobat software. Cleaning and verification and validation were conducted for each report. Each report generated multiple spreadsheets: a spreadsheet for each firearm type reported. Data released by the ATF in 2020 were already in Excel format. Attribute names for each spreadsheet were harmonized for consistency across time periods.

Stata statistical software was used to merge each spreadsheet by RDS (region-district-sequence) key across firearm type and year. Manufacturing and export totals for each year were also calculated in Stata. Data were exported from Stata in table format. Manufacturing total and export totals for all years were calculated in Excel. Percent of total manufactured firearms was also calculated as an aggregate column. Data were sorted by percent of total manufactured firearms. The top 100 locations, which were all locations contributing to more than one percent of the manufactured total, were selected for georeferencing, and stored as a separate spreadsheet.

Pivot table functions in Excel were used to aggregate the number of firearms manufactured by each U.S. state. Table 1 was created by selecting the top manufacturers that contributed to 75 percent of the total firearms manufactured, from 2000-2020. Table 2 was created by aggregating by license name, across multiple locations, to find the total manufactured firearms by licensee. Fig. 1 was created using ArcGIS Pro software. First, the top manufacturing locations were georeferenced with ArcGIS Online geocoding services, based on the address listed by the ATF. The symbol size was changed to represent the quantity of firearm manufactured at each location. The state total table was also joined to U.S. Census Bureau TIGER line files for the U.S. The figure represents a map of the lower 48 states with total firearms production by state and total firearm manufacturing by location.

## Ethics Statements

Our work did not involve human subjects, animal experiments, or social media platforms. Our work is an aggregation of public records and reports.

## CRediT Author Statement

**Lisa Jordan:** Conceptualization, Methodology, Data curation, Validation, Writing; **Marwa Elessway:** Conceptualization, Methodology, Data curation, Validation, Writing; **Graham Munro-Ludders:** Conceptualization, Methodology, Data curation, Validation, Writing.

## Declaration of Competing Interest

The authors declare that they have no known competing financial interests or personal relationships that could have appeared to influence the work reported in this paper. The authors declare the following financial interests/personal relationships which may be considered as potential competing interests.

## Data Availability

U.S. Annual Firearms Manufacturing and Export Report: Aggregate Time Series Dataset 2000-2020 (Original data) (Mendeley Data).
